# Patent Foramen Ovale With Platypnea – Orthodeoxia Syndrome: A Case Report

**DOI:** 10.7759/cureus.10958

**Published:** 2020-10-15

**Authors:** Sabawoon Mirwais, Maiwand Mirwais, Alveena Altaf, Jennifer Collins

**Affiliations:** 1 Internal Medicine, Lahey Hospital and Medical Center, Burlington, USA; 2 Cardiology, Lahey Hospital and Medical Center, Burlington, USA; 3 Internal Medicine-Pediatrics, Memorial Hermann-Texas Medical Center, Houston, USA; 4 Cardiovascular Medicine, Lahey Hospital and Medical Center, Burlington, USA

**Keywords:** orthodeoxia, pfo, platypnea, orthodeoxia syndrome, pfo closure, pos

## Abstract

Platypnea-orthodeoxia syndrome (POS) is an extraordinary medical condition characterized by positional dyspnea (platypnea) and arterial desaturation or hypoxemia (orthodeoxia) in the setting of an upright position. The difficulty breathing is alleviated upon lying down. It is the opposite of orthopnea and is manifested by a decrease in oxygen saturation when changing from supine to an orthostatic position. POS can have an intracardiac or an extracardiac etiology. Herein we report a case of an 87-year-old man presenting with acute on chronic dyspnea who showed promising improvement in oxygen saturation after patent foramen ovale (PFO) closure.

## Introduction

Platypnea-orthodeoxia syndrome (POS) is an extraordinary medical condition characterized by positional dyspnea (platypnea) and arterial desaturation or hypoxemia (orthodeoxia) in the setting of an upright position. The difficulty breathing is alleviated upon lying down [1]. It is the opposite of orthopnea and is manifested by a decrease in oxygen saturation when changing from supine to an orthostatic position [2-5]. The arterial desaturation and hypoxemia are caused by mixing of deoxygenated venous blood with oxygenated arterial blood facilitated by the presence of a cardiopulmonary shunt. POS can have an intracardiac or an extracardiac etiology. Intracardiac causes can include a patent foramen ovale (PFO), atrial septal defect (ASD), atrial septal aneurysm (ASA) with septal fenestration, partial anomalous pulmonary venous connection (PAPVC), transposition of great vessels, and unroofed coronary sinus [1]. Extracardiac causes of POS include pulmonary parenchymal and arteriovenous (AV) malformations [5,6]. The intracardiac POS usually occurs in the setting of a connection between the cardiac chambers enabling right-to-left communication, and this most commonly happens at the inter-atrial level [3]. The most commonly encountered intracardiac cause of a shunt causing POS is a patent foramen ovale [1,6]. The literature being primarily comprised of case reports not only makes under-diagnosis likely but also enables the true incidence of POS to remain masked [5].

## Case presentation

An 87-year-old man with a past medical history significant for chronic obstructive pulmonary disease (COPD), myelodysplastic syndrome (MDS) and subsequent anemia of chronic disease, pulmonary nodule, obstructive sleep apnea (OSA), coronary artery bypass grafting (CABG), and percutaneous coronary interventions (PCI) for coronary artery disease presented with over a year history of exertional dyspnea that had progressed in the last few weeks before his presentation.

**Figure 1:**
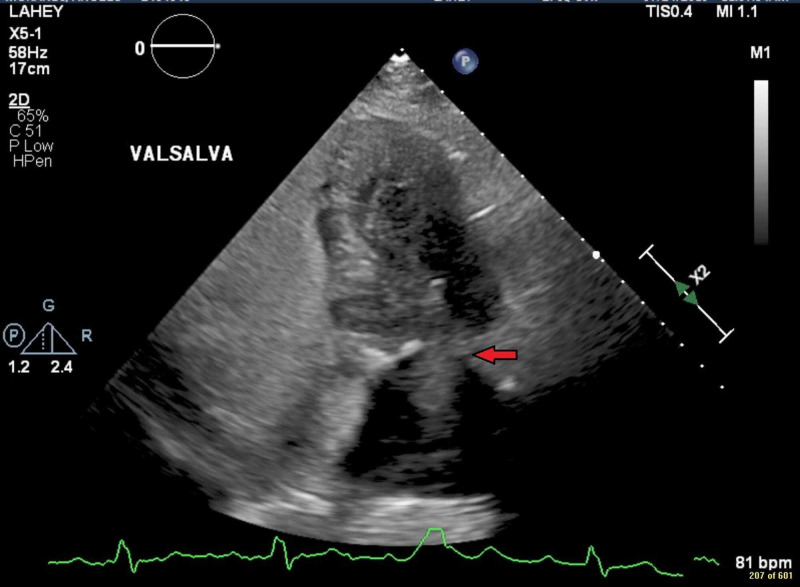
Patent foramen ovale with significant right-to-left inter-atrial shunting while sitting upright with early appearance of left atrial saline contrast (red arrow).

Our patient had been a one pack-per-day smoker for 50 years with a remote past history of occupational asbestos exposure. The first CABG was complicated by disruption of the left phrenic nerve and left-sided diaphragmatic paralysis leading to severe dyspnea. The patient followed in cardiology clinic for his numerous cardiovascular comorbidities. Over a year before the patient’s presentation, he was admitted for dyspnea that was diagnosed as secondary to acute on chronic symptomatic anemia in the setting of recurrent epistaxis with a background of MDS. History of a recent prior admission with symptoms of angina but an unremarkable nuclear stress test prompted the primary team to perform an exercise stress echocardiogram to investigate his dyspnea. The stress test was limited by dyspnea with pulse-oxygen saturation ranging 92%-96% on room air and was negative for both electrocardiographic and echocardiographic evidence of ischemia at the achieved workload. On the echocardiographic imaging part of the test, it was noted that the inter-atrial septum was aneurysmal. A subsequent complete transthoracic echocardiogram (TTE) confirmed that the inter-atrial septum was aneurysmal with no color Doppler evidence of an ASD. The estimated ejection fraction on both these investigations was recorded to be 60%-65%. Over the course of time following this admission, the patient underwent multiple hospitalizations for diverse complaints such as weakness, rectal bleeding, hematuria, urosepsis, syncope, and fever. During this period of time, he also underwent multiple follow-up echocardiograms that showed no significant changes when compared to prior studies. It was only until a month prior to his current presentation that a transesophageal echocardiogram (TEE) with agitated saline contrast revealed a large PFO with a substantial right-to-left inter-atrial shunt and a hypermobile inter-atrial septum (Figure 1). 

On his current presentation, the patient stated that his dyspnea was worse whenever he would be up and moving. He had received two units of packed red blood cells two weeks prior to his admission to the hospital. In the emergency department, his vital signs were noted to be stable, and there was no hypoxia at rest. Laboratory studies were remarkable for a hemoglobin level of 8.2 g/dL, a decrease from approximately two months’ prior hemoglobin level of 9.6 g/dL. Chest x-ray showed no acute cardiopulmonary process at play. Electrocardiogram (EKG) showed no acute ST-T wave changes. The patient was transfused one unit of packed red blood cells and admitted to the hospital medicine floor for further management. Anemia work-up confirmed anemia of chronic disease in the setting of MDS with mild folate deficiency, and he was started on folic acid replacement therapy.

**Figure 2:**
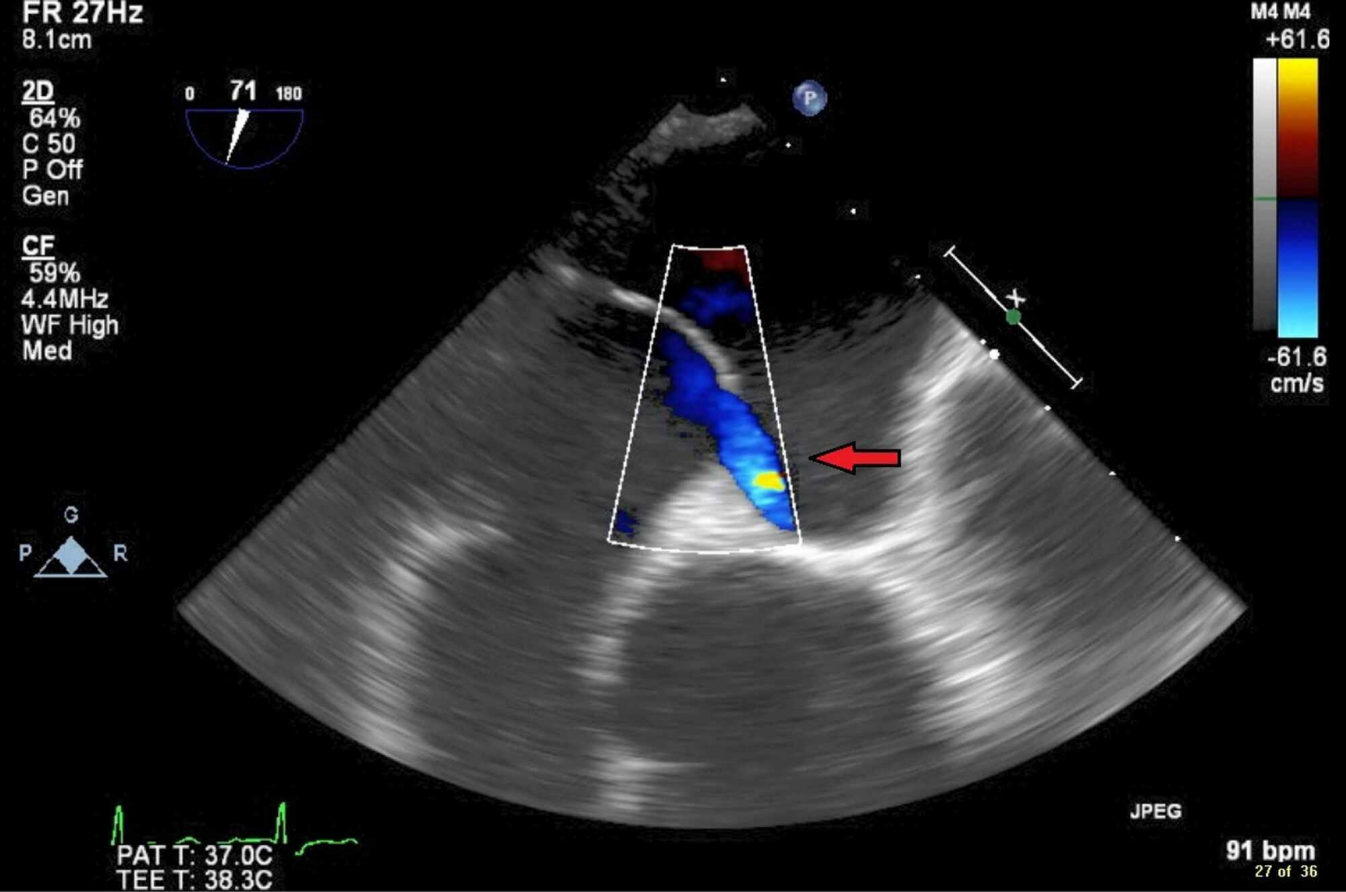
TEE image of PFO demonstrated by color Doppler revealing right-to-left inter-atrial shunting at rest (red arrow). TEE, transesophageal echocardiogram; PFO, patent foramen ovale.

During the course of his admission with multiple episodes of arterial desaturation on ambulation, pulmonology was consulted. On bedside evaluation of the patient by pulmonology, he was noted to have an abrupt oxygen desaturation as checked on a pulse oximeter upon standing to 86% on room air. Significant shunting was evidenced on a bedside shunt study using supplemental oxygen at 100% FiO2, and hypoxemia was noted upon sitting from a supine position. This observation along with the recent TTE findings of a large PFO made the patient a good candidate for further exploration and a possible eventual closure of the PFO. He was subsequently scheduled for a TEE for a better view of his heart and the great vessels. The TEE showed estimated ejection fraction (EF) of 65% and a large PFO with right-to-left inter-atrial shunt (Figure 2).

**Figure 3:**
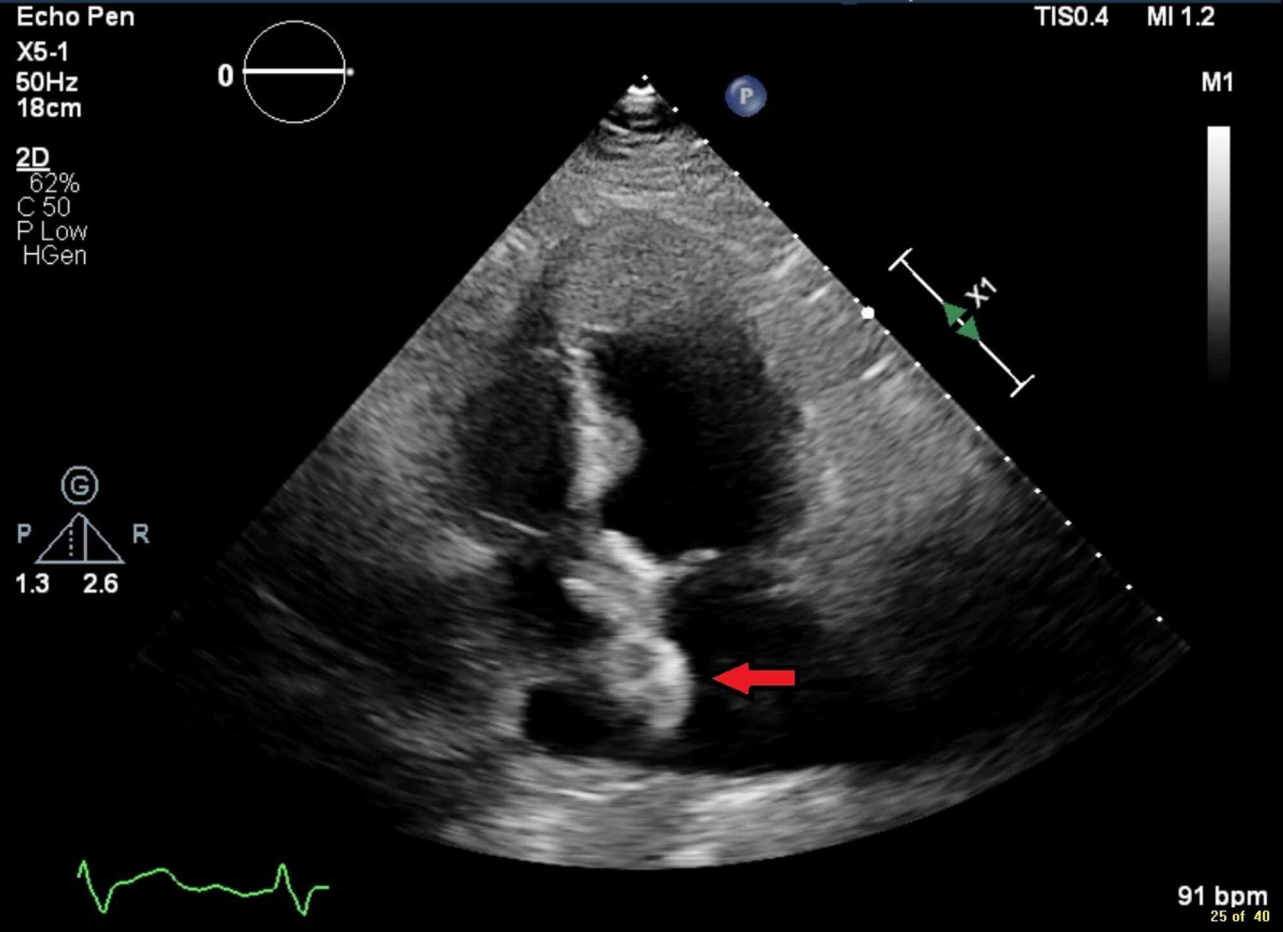
A 25-mm Amplatzer PFO occluder device visualized on a TTE (red arrow). PFO, patent foramen ovale; TTE, transthoracic echocardiogram.

Based on these developments the primary team decided to perform right heart catheterization and PFO closure. He had a 25-mm Amplatzer PFO occluder device placed with intracardiac echocardiographic guidance without any immediate complications. The implanted PFO occluder device was noted to be in stable position with trivial residual inter-atrial shunting on the morning after the procedure (Figure 3). Patient was instructed to follow up with his cardiologist within two weeks of discharge. His symptom of positional dyspnea improved dramatically, and he stopped having any changes in his blood oxygen saturation with different positions of the body. And on multiple occasions post-PFO closure, his pulse oximeter reading was never noted to be below 96%. 

## Discussion

Following birth, the septum primum and septum secundum normally fuse to give rise to an intact inter-atrial septum; but in approximately 25% of the worldwide adult population, the fusion does not occur, and the septum primum and secundum are only juxtaposed [7,8]. Isolated PFO does not cause significant right-to-left inter-atrial shunting as the higher left atrial pressure allows functional closure of the PFO.

Normally, blood from the superior vena cava (SVC) flows down to the anterior right atrium and blood from the inferior vena cava (IVC) flows up to the posterior right atrium. An altered cardiac anatomy can encourage the blood from the IVC to flow directly into the left atrium via the PFO or ASD. With absent right atrial hypertension, right-to-left shunting across PFO, ASD, or fenestrated atrial septal aneurysm (ASA) has been noticed to occur in the presence of a concomitant secondary cardiac or pulmonary functional anomaly [1]. This redirection of the blood into the left atrium is proposed to occur due to the repositioning of inter-atrial septum in the orthostatic position. This repositioning or increased mobility of the inter-atrial septum giving rise to intracardiac POS can be observed in aortic aneurysm, aortic root dilatation/elongation in individuals with an ASA, or in persistent Eustachian valve or Chiari’s network [9-12]. In the setting of tricuspid regurgitation, a jet of blood can be projected to the left atrium through the inter-atrial shunt [13-15]. The ascending aorta and inter-atrial septum can be more horizontal in the setting of osteoporosis-related kyphoscoliosis [16,17].

Pulmonary causes of POS include primary pulmonary arteriovenous malformations (AVMs), advanced hepatopulmonary syndrome (HPS), and other conditions causing pulmonary arteriovenous shunting such as parenchymal lung diseases and interstitial lung disease or consolidation with primary involvement of the lung bases and consequent severe ventilation/perfusion (V/Q) mismatch [5,18].

POS can also occur due to combined cardiopulmonary aberration. The perfect example of such an aberration is lung resection. Lung resection decreases the pulmonary vascular bed area, which when combined with commonly co-existing chronic hypoxemia can result in increased pulmonary vascular resistance and right ventricular afterload as well as reduced right ventricular compliance. Post-pneumonectomy fluid overload can also contribute to an increasing right ventricular afterload, and all these factors converge into an elevated right atrial pressure allowing right-to-left inter-atrial shunt [2,19]. 

Given our patient's complex past medical history, it was rather difficult to get to the final diagnosis of POS caused by a large PFO, and he was treated for acute COPD exacerbation instead during the weeks prior to this presentation. This case reiterates the importance of having a high index of suspicion in challenging cases where the standard therapy has failed to alleviate the symptoms.

## Conclusions

POS, in the background of scarce literature, remains an intriguing clinical conundrum to solve. Most of the available case reports involve the elderly. The prevalence of other diagnosed medical conditions in this population, relative to the younger age groups, is high. It is understandable if POS is not high on the list of differential diagnoses for dyspnea. But considering the fact that a simple closure of the inter-atrial shunt, such as in the case of our patient, can point things in the right direction, POS should be a condition that one should always rule out. A thorough history and physical examination, the backbone of Medicine, take significant precedence when dealing with patients suspected to have POS. 
